# Curative Effect of Heat-sensitive Moxibustion on Primary Dysmenorrhea: A Meta-Analysis

**DOI:** 10.1155/2022/1281336

**Published:** 2022-07-30

**Authors:** Ningning Xu, Yingjie Huang, Hai Huang, Yuxin Huang, Siran Lai, Zhenyu Zhang, Yizheng Zhong

**Affiliations:** ^1^The First Clinical Medical College, Guangzhou University of Chinese Medicine, Guangzhou 510405, Guangdong, China; ^2^Clinical Medical College of Acupuncture Moxibustion and Rehabilitation, Guangzhou University of Chinese Medicine, Guangzhou 510405, Guangdong, China; ^3^The Second Clinical Medical College, Guangzhou University of Chinese Medicine, Guangzhou 510405, Guangdong, China; ^4^The Fifth Clinical Medical College, Guangzhou University of Chinese Medicine, Guangzhou 510405, Guangdong, China; ^5^Guangzhou Women and Children Medical Center, Guangzhou 510405, Guangdong, China

## Abstract

**Background:**

Primary dysmenorrhea (PD) refers to functional dysmenorrhea, typically characterized by cyclical, pronounced lower abdominal pain and seriously affects a woman's work and quality of life. Some studies have reported that heat-sensitive moxibustion (HSM) is expected to alleviate the clinical symptoms. This systematic review aimed to evaluate the current evidence regarding the efficacy and safety of HSM on PD.

**Methods:**

7 databases including PubMed, Embase, Cochrane Library, Web of Science, China National Knowledge Infrastructure (CNKI), Wan Fang Data Knowledge Service Platform (Wan Fang Data), and China Science and Technology Journal Database (VIP) were searched for clinical randomized controlled trials. Meanwhile, Revman 5.3 software was used to evaluate the methodological quality of the included literature. The confidence interval (CI) of either relative risk or mean difference was set to 95%. Besides, the heterogeneity of the research results is tested by I^2^.

**Results:**

19 studies were ultimately included in this meta-analysis. All of them were declared as random controlled trials. 18 studies reported the total effective rate of the test group and the control group, which was significantly higher (RR: 0.92; 95% CI: 0.85,0.99; *P*=0.031 < 0.05) than the control group. It is demonstrated that the VAS score of the test group, totally 9 studies included, was significantly lower (SMD: −0.98; 95% CI: −1.15, −0.81; *P* < 0.001). The meta-analysis of 6 studies showed the symptom score of the test group was significantly lower (SMD: −0.67; 95% CI: −0.87, −0.47; *P* < 0.001). There were the CMSS results of 3 studies which were significantly lower (SMD: −0.88; 95% CI: −1.13, −0.62; *P* < 0.001). Combined with the results of subgroup analysis, compared with the control group, the test group had advantages in the VAS score, symptom score, and CMSS score.

**Conclusions:**

The result has revealed the effectiveness and feasibility of HSM in treating PD, especially in improving the total effective rate and reducing the VAS score, symptom score, and CMSS score.

## 1. Introduction

Primary dysmenorrhea (PD) refers to functional menstrual pain, which is distinguished from secondary dysmenorrhea with pelvic organic lesions [1,2]. The typical symptom of PD is periodic and obvious lower abdominal pain that occurs around menstruation [3]. Possible concomitant symptoms include involved pain in the waist and sacrum, diarrhea, nausea and vomiting, fatigue, mood disorders, and syncope in severe cases. [4] The pathogenesis of PD is closely related to the increased secretion of prostaglandin F2*α* (PGF2*α*) and prostaglandin E2(PGE2) in the late luteal phase, as well as the contraction of the smooth muscle and the blood vessels [5]. PD is a common gynecological disease. A retrospective analysis based on large community samples [6] indicates that the prevalence of PD rages from 16% to 91% among women of childbearing age. Other studies suggest that the worldwide prevalence of PD may be higher, ranging from 45% to 95% [1]. PD has a general and obvious adverse effect on women's daily life, work, and study [7]. The treatment of PD includes drug therapies, such as non-steroidal anti-inflammatory drugs (NSAIDs), hormones, as well as complementary and alternative therapies, such as moxibustion, acupuncture, and local hot compress [3]. Due to their high benefit, NSAIDs are recommended as the clinical first-line drugs of PD and have been widely used at present [5]. However, a systematic review [8] shows that NSAIDs used for PD may cause certain adverse reactions, including gastrointestinal symptoms (such as nausea and vomiting) and nervous system disorders (such as dizziness, headache, drowsiness, and exertion). Besides, NSAID resistance in PD is also common and worth paying attention to [9]. Thus, it may be of practical significance to find a treatment with definite curative effect and satisfactory safety to reduce the dosage of NSAIDs or replace them.

uterine microcirculation, so as to relieve pain. In addition, a double-arm-designed RCT [11] reported that the curative effect of moxibustion was similar to that of NSAIDs (ibuprofen-sustained release capsule) in the treatment of PD, and there was no significant adverse event. Heat-sensitive moxibustion (HSM) is a moxibustion therapy invented by Professor Chen RX, and it focuses on “heat-sensitive” sensation of acupoints as well as Deqi of moxibustion [12, 13]. HSM pays great attention to standardizing the clinical practice of moxibustion, and meanwhile it pays great attention to the individual differences of patients [12]. According to a prospective cohort study based on propensity score match [14], HSM stimulates heat-sensitized acupoints with a better effect than traditional moxibustion as a treatment of PD. In addition, a case [15] reported that by standardizing the moxibustion temperature, dose, and treatment time, HSM effectively relieved menstrual pain. However, as a complementary and alternative therapy for PD, the clinical efficacy and safety of HSM still need to be systematically studied. Therefore, this study aims to provide as much evidence as possible through meta-analysis for the feasibility of HSM in treating PD.

## 2. Methods

### 2.1. Inclusion Criteria

The population-intervention Comparators-Outcomes-Study design (PICOS) framework was used as the eligibility criteria for the review as follows.

#### 2.1.1. Selection of Studies

All randomized controlled trials (RCTs) investigating HSM combined with other therapies in the treatment of PD were not limited by language or publication status.

#### 2.1.2. Selection of Participants

The study subjects were female patients with clear clinical diagnostic criteria and all included patients were clearly diagnosed with PD.

#### 2.1.3. Types of Interventions

The experimental group was treated with HSM for intervention, and the control group was treated with non HSM, including NSAIDs, acupuncture therapy, moxibustion, progesterone, and traditional Chinese medicine. Studies that did not meet the above inclusion criteria were excluded. In addition, the following exclusion criteria were applied: ①non-randomized controlled trial research literature; ②participants were non-primary dysmenorrhea patients; ③literature without original data or incomplete research data; and ④literature on interventions that did not meet the inclusion criteria.

### 2.2. Types of Outcome Measures

According to the author's definition, we found that the commonly used evaluation indicators include the total effective rate, cox menstrual symptom scale (CMSS), visual analogue scale (VAS) score, and symptom score [16].

### 2.3. Data Sources and Search Strategy

Computer searches of PubMed, Embase, Cochrane Library, Web of Science, China National Knowledge Infrastructure (CNKI), Wan Fang Data Knowledge Service Platform (Wan Fang Data), and China Science and Technology Journal Database (VIP) were conducted, all from the establishment of the database to January 2022. The search strategy involved the use of the following keywords: “Heat-sensitive Moxibustion,” “thermal moxibustion,” “Dysmenorrhea” “ dysmenorrhoea,” “HSM,” “Menstrual Pain,” “Primary dysmenorrhea,” etc. Taking PubMed as an example, the search terms and strategies are as follows: (((thermal moxibustion[Title/Abstract]) OR (Heat-sensitive Moxibustion[Title/Abstract])OR (HSM[Title/Abstract]))) AND (Dysmenorrhea[mesh] OR dysmenorrhea[tiab] OR “Pain, Menstrual”[tiab] OR “Menstrual Pain”[tiab] OR “Menstrual Pains”[tiab] OR “Pains, Menstrual”[tiab] OR “Menstruation, Painful”[tiab] OR “Menstruations, Painful”[tiab] OR “Painful Menstruation”[tiab] OR “Painful Menstruations”[tiab] OR “Primary dysmenorrhea”[tiab]) AND (((clinical[tiab] AND trial[tiab]) OR “clinical trials as topic”[mesh] OR “clinical trial”[pt] OR random*∗*[tiab] OR “random allocation”[mesh] OR “therapeutic use”[sh]))). The search strategies of other databases follow their search rules.

### 2.4. Literature Screening and Data Extraction

Two researchers conducted literature screening independently in strict accordance with inclusion and exclusion criteria, and managed and identified the retrieved literature by Note Express software. Excel software was used to establish the database of literature information extraction, including research types, number of cases, diagnostic criteria, intervention and treatment, methodology and curative effect of standard, outcome indicators, and adverse reactions. Finally, the results were cross-checked, and disputes were resolved through discussion or seeking the opinions of the third party.

### 2.5. Quality Assessment

According to the Cochrane system assessment handbook that provides the standard, we adopted Revman5.3 for the methodological quality evaluation of the literature. Risk items include random sequence, allocation hiding, blindness, integrity of outcome data, risk of selective reporting bias, and other biases. If each of the 7 items is assessed as low risk, the study bias risk is assessed as low risk. If one or more entries are assessed as high risk, the study is assessed as high risk.

### 2.6. Statistical Analysis

Stata 15.1 software was used to perform the meta-analysis. If relative risk (RR) is used for dichotomous variables, the confidence interval (CI) is set at 95%. Continuous variables were represented by STD mean difference (SMD), and confidence interval (CI) was set at 95%. Heterogeneity of the research results was tested by I^2^. If I^2^ ≤ 50%, the outcome data of the fixed effects model (FE) were selected for analysis; if I^2^ > 50%, the outcome data of the random effects model (RE) were selected for reference analysis. Sensitivity analysis was used to analyze the sources of heterogeneity and to assess the stability of the meta-analysis results.

## 3. Result

### 3.1. Search Results

**Figure 1:**
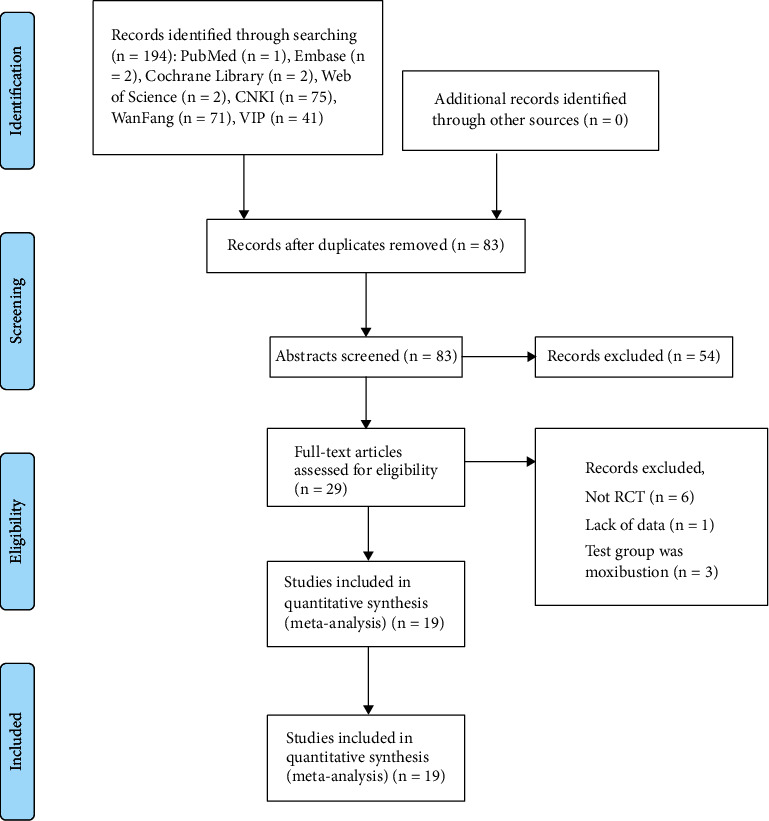
The inclusion process of literature.

Acting by the search strategy, 194 references were identified. After excluding duplicate studies, 83 studies were scanned based on their abstracts and titles. Then, 29 articles were evaluated by full text. After full-manuscript assessment, 10 records were excluded with the following reasons: not RCT (*n* = 6), lack of outcomes (*n* = 1), and the test group was manual acupuncture treatment (*n* = 3). Eventually, 19 studies [17–35] were included in this meta-analysis (Table 1). The PRISMA statement flow chart shows this process (Figure 1).

### 3.2. Heat-Sensitive Acupoints

The heat-sensitive acupoints and their frequency and positions were included in this meta-analysis (Tables 2 and 3). Data analysis of the 19 heat-sensitive acupoints collated from the 19 research studies included in this study for the treatment of primary dysmenorrhea involved 14 heat-sensitive points (Baliao (BL31, BL32, BL33, BL34) contained 4 acupuncture points, containing Ciliao (BL32)). The top 4 heat-sensitive points used in frequency were Guanyuan (CV4), Sanyinjiao (SP6), Zigong (EX-CA1), and Ciliao (BL32).

### 3.3. Risk of Bias Assessment

**Figure 2:**
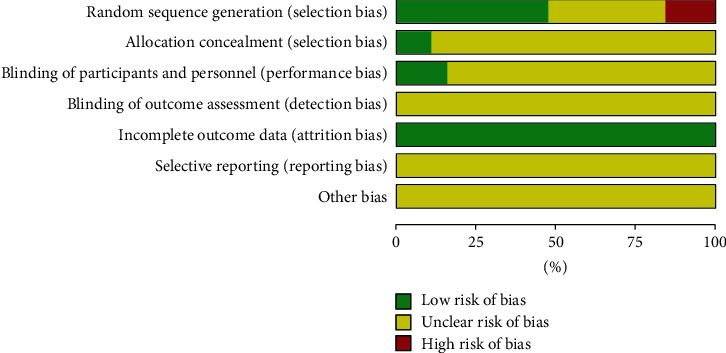
Risk of bias assessment in studies.

**Figure 3:**
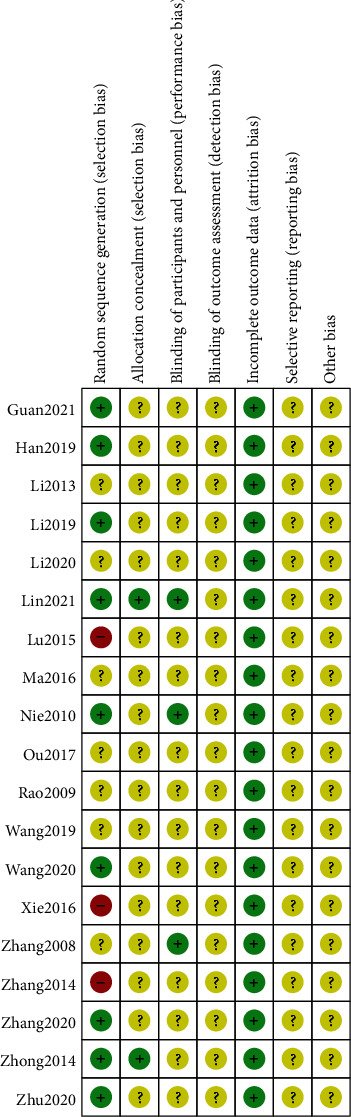
Risk of bias assessment for each included study in the review.

Nineteen included studies [17–35] involved two-arm designs, and 9 trials reported proper generation methods (random number table) with a low risk of bias [17–19, 21–23, 25, 31, 33] (Figures 2 and 3). Seven trials did not describe the randomization procedure clearly [20, 24, 26, 27, 32, 34, 35]. Three trials reported generation methods (order of treatment or therapeutic measures) with a high risk of bias [28–30]. Three trials reported the blinding of participants and personnel [18, 33, 35]. Two trials reported any allocation concealment [18, 31]. Incomplete outcome data, selective reporting, and other biases had a low risk of bias. None of the trials reported the blinding of outcome assessment.

### 3.4. Primary Outcomes

#### 3.4.1. Total Effective Rate

**Figure 4:**
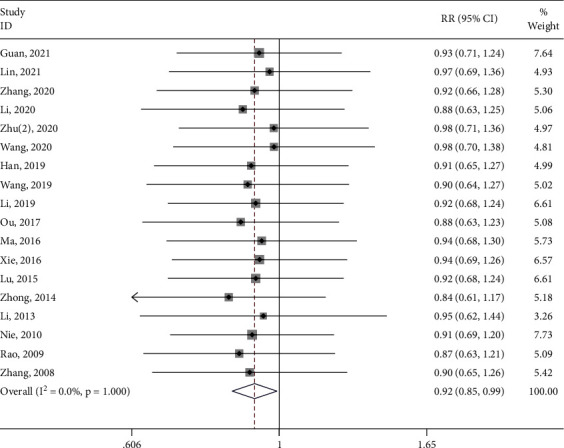
Forest plot of the total effective rate.

Eighteen studies [17–29, 31–35] reported the total effective rate of the test group and the control group. The meta-analysis showed that the total effective rate of the test group was significantly higher (RR: 0.92; 95% Cl: 0.85,0.99; *P*=0.031 < 0.05, I^2^ = 0%, Figure 4) than the control group. I^2^ = 0% showed that the meta-analysis of total effective rate has high stability. Subgroup analysis (Supplement Figure 1) was performed for the type of control intervention (Traditional Chinese medicine, Ibuprofen, Moxibustion, Progesterone, and Acupuncture). The meta-analysis showed that the result of control intervention was low heterogeneity (I^2^ = 0%), and the total effective rate of the test group was not significantly higher than the control group (*P* > 0.05).

#### 3.4.2. VAS Score

**Figure 5:**
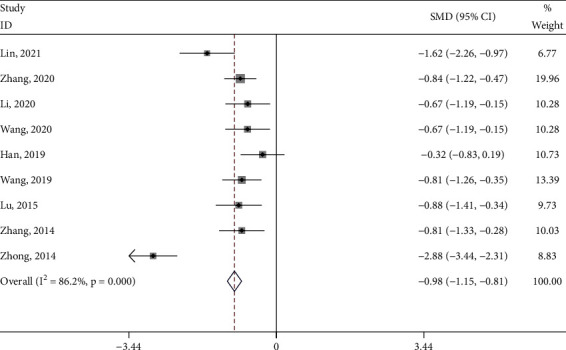
Forest plot of the VAS Score.

**Figure 6:**
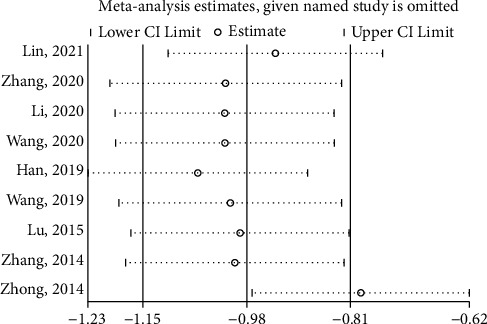
Sensitivity analysis of the VAS Score for each included study in the review.

**Figure 7:**
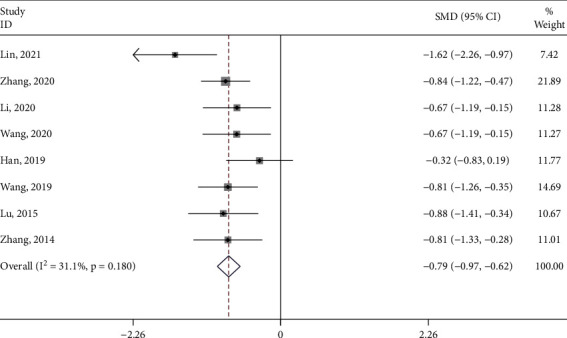
Forest plot of the VAS Score after excluding one trail.

Nine studies [18–20, 22–24, 29–31] reported the VAS score of the test group and the control group. The meta-analysis showed that the VAS score of the test group was significantly lower (SMD: -0.98; 95% Cl: -1.15,-0.81; *P* < 0.001, I^2^ = 86.2%, Figure 5) than the control group. The results of all these trials showed high heterogeneity, and thus a sensitivity analysis was conducted (Figure 6), which showed that the included trail [31] had a more significant impact on the results. A careful review of the included trail [31] found that the intervention in the control group was ginger-partitioned moxibustion or patients were limited to cold-dampness coagulation syndrome. The remaining eight studies were used to analyze the VAS score and get new results (SMD: −0.79; 95% Cl: −0.97,−0.62; *P* < 0.001, I^2^ = 31.1%, Figure 7). Subgroup analysis (Supplement Figure 2) was performed for the type of control intervention (Traditional Chinese medicine, Ibuprofen, and Moxibustion). The meta-analysis showed that the result of control interventions (Ibuprofen, Moxibustion) was high heterogeneity (I^2^ = 62.1% and I^2^ = 94.9%), the result of control interventions (Traditional Chinese medicine) was low heterogeneity (I^2^ = 0%), and the VAS Score of the test group was significantly higher than the control group. (*P* < 0.05).

#### 3.4.3. Symptom Score

**Figure 8:**
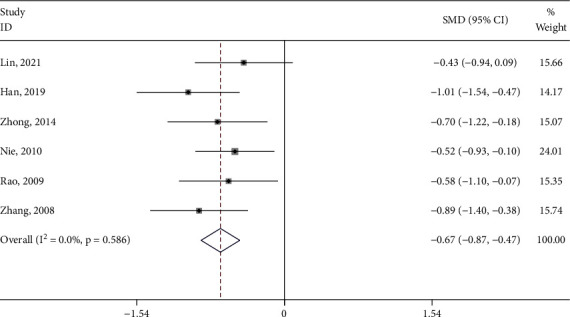
Forest plot of the symptom score.

Six studies [20, 23, 31, 33, 34] reported the symptom score of the test group and the control group. The meta-analysis showed that the symptom score of the test group was significantly lower (SMD: -0.67; 95% Cl: -0.87,-0.47; *P* < 0.001, I^2^ = 0.0%, Figure 8) than the control group. Subgroup analysis (Supplement Figure 3) was performed for the type of control intervention (Traditional Chinese medicine, Ibuprofen, Moxibustion, and Acupuncture). The meta-analysis showed that the result of control interventions was low heterogeneity (I^2^ = 0%), and the symptom score of the test group was significantly higher than the control group (*P* < 0.05).

#### 3.4.4. CMSS Score

**Figure 9:**
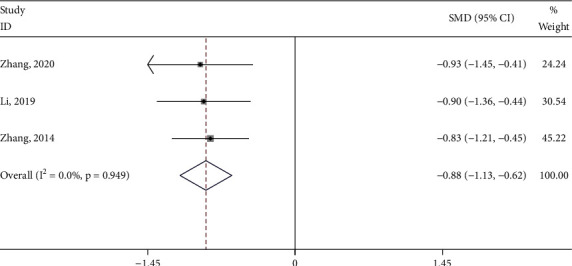
Forest plot of the CMSS score.

Three studies [19, 25, 30] reported the CMSS score of the test group and the control group. The meta-analysis showed that the CMSS score of the test group was significantly lower (SMD: −0.88; 95% Cl: −1.13,−0.62; *P* < 0.001, I^2^ = 0.0%, Figure 9) than the control group. Subgroup analysis (Supplement Figure 4) was performed for the type of control intervention (Moxibustion, Progesterone). The meta-analysis showed that the result of control interventions was low heterogeneity (I^2^ = 0%), and the CMMS score of the test group was significantly higher than the control group (*P* < 0.05).

#### 3.4.5. Publication Bias

**Figure 10:**
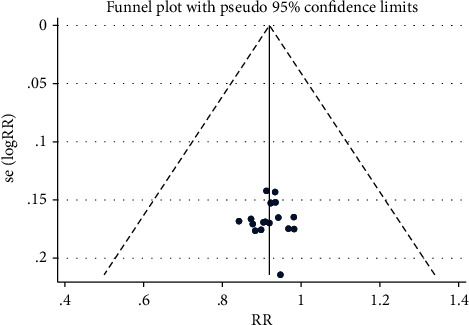
Funnel plot of the total effective rate.

The funnel plot (Figure 10) of the total effective rate was symmetrically distributed. What's more, Eggr's test showed no potential publish bias (*P*=0.976).

## 4. Discussion

Primary dysmenorrhea [36] is defined as dysmenorrhea that occurs in the absence of pelvic pathology. Dysmenorrhea usually begins after the ovulatory cycle is established during puberty. The associated pain is caused by the excessive release of prostaglandins (PGs) during the shedding of the endometrium. High levels of PGs during menstruation cause excessive contraction of the uterine smooth muscle, leading to hypoxia and ischemia, producing a painful sensation. NSAIDs can be used as a first-line treatment for PD; it is uncertain that NSAIDs are the best clinical choice for the treatment of PD [37]. These drugs reduce uterine tone and contractility by blocking cyclooxygenase to reduce prostaglandin synthesis, thereby relieving prostaglandin-induced spastic contractions of the uterus and bringing relief from dysmenorrhea. However, long-term administration can cause serious gastrointestinal, cardiovascular, skeletal, and renal adverse effects [38]. Therefore, it is important to seek new therapies with few adverse effects and low side effects for the treatment of PD. Some studies have reported that HSM is expected to alleviate the clinical symptoms. Therefore, this study aimed to provide as much evidence as possible for the feasibility of HSM in the treatment of PD through a meta-analysis.

HSM as one of the suspension therapies [12] is a new therapy to improve the efficacy of moxibustion, which uses the moxa heat generated by burning moxa velvet to apply moxibustion to heat-sensitive acupuncture points, stimulating the body's acupuncture points to produce moxibustion sensation. When moxibustion is applied to heat-sensitive acupoints, patients experience unusual feelings (referred to as “heat-sensitive sensations”), including penetrating heat, expanding heat, heat transfer, distant heat, deep heat, and other feelings unrelated to heat (such as soreness, bloating, and numbness) [39]. The heat-sensitive phenomenon of moxibustion is one of the manifestations in the activated functional activities of meridian of Chinese medicine just like the arrival of the qi caused by acupuncture stimulation. It is also a sign of activation of the human endogenous functional regulative system [40]. It is a kind of external therapy for internal disorders, directly acting on the pathogenesis, strengthening the antipathogenic qi [41]. The current clinical and experimental research indicate that moxibustion improves the body's immunity and effectively inhibits inflammatory responses [42]. In addition, a study [10] on the mechanism of moxibustion found that moxibustion plays a therapeutic role through its four mechanisms of action: heat, light, moxa smoke, and drug effects. The mechanism of moxibustion treatment for primary dysmenorrhea focuses on adjusting endocrine hormones, regulating immune function and neuro-related factors, and improving uterine microcirculation. Another study [43]concluded that it can also diffuse inhibitory substances in the cerebral cortex, reduce the excitability of the nervous system, and exert sedative and analgesic effects. Compared to oral medications such as NSAIDs and contraceptives, HSM as an external treatment is safer and cheaper, as mugwort is a very common and inexpensive herbal remedy. In conclusion, compared to other treatments, heat-sensitive moxibustion has obvious advantages in terms of efficacy, safety, patient acceptance, and low treatment cost.

The results of this meta-analysis showed that the overall efficiency of HSM for PD was better than the control groups, and HSM was also better than other therapies in terms of improving the CMSS scores, VAS scores, and symptom scores. Six studies [19, 26, 28, 30, 32, 35] suggest that HSM is more effective than traditional moxibustion, four studies [18, 20, 27, 29] suggest that HSM is more effective than oral ibuprofen in the treatment of PD, one study [33] suggests that HSM is more effective than acupuncture combined with massage, and one study [31] suggests that HSM is more effective than ginger moxibustion in the treatment of PD. Combined with the results of subgroup analysis, compared with the control group, HSM had advantages in VAS score, symptom score, and CMSS score. It can be seen that HSM has obvious advantages in the treatment of PD and is worth promoting in the clinics, and clinicians can prefer HSM for PD according to the actual situation. This study included a total of 19 research studies and only 5 had safety analyses that mentioned adverse effects. None of the HSM groups had any adverse effects, which may mean that HSM is safe overall.

There are some limitations in this study. First of all, the 19 RCTs included involved 1325 patients. The overall sample size was not very large. Secondly, the frequency and duration of treatment regimen interventions were not uniform in this study, which affected the accuracy of the results to some extent. Besides, the included studies were all observed for immediate efficacy, and the long-term efficacy of HSM on PD needs to be studied in a progressive manner.

## 5. Conclusions

The findings of this study suggest that heat-sensitive moxibustion was an effective intervention for reducing the VAS score and CMSS score, reducing the dysmenorrhea symptom score, and improving the total effective rate. HSM avoids the disadvantages of systemic medication, which finds it difficult to reduce the disease, and is simple and easy to implement, which is worth further clinical exploration and promotion. This study provides a reference for clinicians in the treatment of primary dysmenorrhea in order to provide data support for the future widespread use of HSM as a community or family self-treatment modality. However, due to the possibility that the randomization bias was high, it is necessary to use a large sample size, multicenter, low bias risk clinical research and basic medical research in the future based on strict control of the research design.

## Figures and Tables

**Table 1:** The basic characteristics of the included studies. T: trial group; C: control group; NA: not reported; ①Total effective rate; ②VAS score; ③Symptom score; and ④CMSS score.

Trail	Sample Size (T/C)	Age (y), Mean ± SD or Median (Range)		Duration		T	C	Main Outcomes	Follow-up time/month
		T	C	T	C				
Guan, 2021	47/46	20.73 ± 5.46	21.41 ± 5.92	5.43 ± 1.22m	5.19 ± 1.38m	HSM	Traditional Chinese medicine	①④	—
Lin, 2021	30/31	23.70 ± 2.78	24.03 ± 2.63	7.60 ± 2.58	8.48 ± 2.57	HSM	Sustained-Release ibuprofen capsules	①②③	3
Zhang, 2020	32/32	18.56 ± 2.35	17.22 ± 3.39	12.46 ± 4.35 m	11.96 ± 4.25m	HSM	Moxibustion	①②④	3
Li, 2020	30/30	23.5	25.1	22.8	4.6	HSM	Sustained-Release ibuprofen capsules	①②	—
Zhu, 2020	31/31	22.97 ± 4.04	21.97 ± 3.63	4.26 ± 2.68	3.09 ± 2.63	HSM	Traditional Chinese medicine	①	3
Wang, 2020	30/30	24.57 ± 3.88	23.73 ± 2.92	7.27 ± 3.86	6.90 ± 3.62	HSM	Traditional Chinese medicine	①②	3
Han, 2019	30/30	NA	NA	NA	NA	HSM	Traditional Chinese medicine	①②③	—
Wang, 2019	30/30	24.4	24.4	4m-13 y	4m-13y	HSM	Traditional Chinese medicine	①②	3
Li, 2019	40/40	22.93 ± 3.41	22.65 ± 3.27	9.43 ± 3.59m	9.06 ± 3.62m	HSM	Progesterone	①④	1
Ou, 2017	30/30	NA	NA	NA	NA	HSM	Moxibustion	①	—
Ma, 2016	35/35	30.0 ± 4.5	30.2 ± 5.0	2.5 ± 0.5	3.0 ± 0.5	HSM	Sustained-Release ibuprofen capsules	①	—
Xie, 2016	40/40	19.3 ± 1.2	19.2 ± 1.3	10.7 ± 2.3 m	10.9 ± 2.5m	HSM	Moxibustion	①	—
Lu, 2015	40/40	14-30	15-29	8m-7 y	6m-6.5y	HSM	Sustained-Release ibuprofen capsules	①②	—
Zhang, 2014	61/56	22.76 ± 4.26	23.30 ± 4.51	6.76 ± 2.26	7.03 ± 2.71	HSM	Moxibustion	②④	3
Zhong, 2014	30/30	23.774 ± 2.582	22.900 ± 2.537	6.67 ± 2.264	7.000 ± 1.894	HSM	Moxibustion	①②③	3
Li, 2013	20/20	20-42	20-42	1-5y	1-5y	HSM	Moxibustion	①	—
Nie, 2010	47/46	19.25 ± 4.03	19.24 ± 3.76	3.02 ± 1.38	2.93 ± 1.27	HSM	Acupuncture	①③	—
Rao, 2009	30/30	22.07 ± 4.50	21.47 ± 4.8	3.65 ± 2.75	3.51 ± 2.99	HSM	Sustained-Release ibuprofen capsules	①③	—
Zhang, 2008	33/32	NA	NA	NA	NA	HSM	Moxibustion	①③	—

**Table 2:** The heat-sensitive acupoints of the included studies. NA: not reported.

Trail	Heat-Sensitive Acupoints
Guan, 2021	Guanyuan (CV4)
Lin, 2021	Guanyuan (CV4), Zhongji (CV3), Zigong (EX-CA1), Qihai (CV6), Sanyinjiao (SP6),
Zhang, 2020	Guanyuan (CV4), Zhongji (CV3), Zigong (EX-CA1)
Li, 2020	Sanyinjiao (SP6)
Zh-, 2020	Guanyuan (CV4), Zigong (EX-CA1), Ciliao (BL32), Sanyinjiao (SP6)
Wang, 2020	Guanyuan (CV4), Zigong (EX-CA1), Ciliao (BL32), Sanyinjiao (SP6), Shenque (CV8)
Han, 2019	Guanyuan (CV4), Zigong (EX-CA1)
Wang, 2019	NA
Li, 2019	Guanyuan (CV4), Zhongji (CV3), Zigong (EX-CA1), Guilai (ST29), Ciliao (BL32), Sanyinjiao (SP6)
Ou, 2017	Guanyuan (CV4), Zhongji (CV3), Zigong (EX-CA1), Sanyinjiao (SP6), Ciliao (BL32)
Ma, 2016	Guanyuan (CV4), Sanyinjiao (SP6), Ciliao (BL32)
Xie, 2016	Guanyuan (CV4)
Lu, 2015	Guanyuan (CV4), Zhongji (CV3), Sanyinjiao (SP6)
Zhang, 2014	Guanyuan (CV4)
Zhong, 2014	Guanyuan (CV4), Zigong (EX-CA1), Ciliao (BL32), Sanyinjiao (SP6)
Li, 2013	Guanyuan (CV4), Zigong (EX-CA1), Sanyinjiao (SP6), Qihai (CV6), Diji (SP8), Shiqizhui (EX-B 8), Bajiao (BL31, BL32, BL33, BL34)
Nie, 2010	Guanyuan (CV4), Zhongji (CV3), Sanyinjiao (SP6)
Rao, 2009	Guanyuan (CV4), Zhongji (CV3), Sanyinjiao (SP6), Ciliao (BL32)
Zhang, 2008	Guanyuan (CV4), Sanyinjiao (SP6)

**Table 3:** Frequency and position of the heat-sensitive acupoints.

Number	Heat Sensitive Acupoints	Frequency (times)	Percentage%	Position
1	Guanyuan (CV4)	17	27.87	Abdomen
2	Sanyinjiao (SP6)	13	21.31	lower extremities
3	Zigong (EX-CA1)	9	14.75	Abdomen
4	Ciliao (BL32)	8	11.67	Sacrum
5	Zhongji (CV3)	7	13.11	Abdomen
6	Qihai (CV6)	2	3.28	Abdomen
7	Shenque (CV8)	1	1.64	Abdomen
8	Baliao (BL31, BL32, BL33, BL34)	1	1.64	Sacrum
9	Diji (SP8)	1	1.64	lower extremities
10	Shiqizhui (EX-B 8)	1	1.64	Sacrum
11	Guilai (ST29)	1	1.64	Abdomen

## Data Availability

All available data are included in this manuscript.

## Supplementary Materials

Supplementary Materials Subgroup analysis of the total effective rate was performed on Supplemental Figure 1. Subgroup analysis of the VAS score was performed on Supplemental Figure 2. Subgroup analysis of the symptom score was performed on Supplemental Figure 3. Subgroup analysis of the CMSS score was performed on Supplemental Figure 4.

## References

[1] Bernardi M., Lazzeri L., Perelli F., Reis F. M., Petraglia F. (2017). Dysmenorrhea and related disorders. *F1000Research*.

[2] Santos L. B. D., Barbosa I. R., Dantas T. H. D. M. (2022). Prevalence of primary dysmenorrhea and associated factors in adult women. *Revista da Associação Médica Brasileira (1992)*.

[3] Burnett M., Lemyre M. (2017). No. 345-Primary dysmenorrhea consensus guideline. *Journal of Obstetrics and Gynaecology Canada*.

[4] Karout S., Soubra L., Rahme D., Karout L., Khojah H. M. J., Itani R. (2021). Prevalence, risk factors, and management practices of primary dysmenorrhea among young females. *BMC Women’s Health*.

[5] Itani R., Soubra L., Karout S., Rahme D., Karout L., Khojah H. M. J. (2022). Primary dysmenorrhea: pathophysiology, diagnosis, and treatment updates. *Korean Journal of Family Medicine*.

[6] Ju H., Jones M., Mishra G. (2014). The prevalence and risk factors of dysmenorrhea. *Epidemiologic Reviews*.

[7] Sharghi M., Mansurkhani S. M., Larky D. A. (2019). An update and systematic review on the treatment of primary dysmenorrhea. *JBRA Assist Reprod*.

[8] Marjoribanks J., Ayeleke R. O., Farquhar C., Proctor M. (2015). Nonsteroidal anti-inflammatory drugs for dysmenorrhoea. *Cochrane Database of Systematic Reviews*.

[9] Oladosu F. A., Tu F. F., Hellman K. M. (2018). Nonsteroidal antiinflammatory drug resistance in dysmenorrhea: epidemiology, causes, and treatment. *American Journal of Obstetrics and Gynecology*.

[10] Pan S., Wang S., Li J. (2022). Moxibustion for primary dysmenorrhea: an adjuvant therapy for pain relief. *Evidence-based Complementary and Alternative Medicine*.

[11] Yang M., Chen X., Bo L. (2017). Moxibustion for pain relief in patients with primary dysmenorrhea: a randomized controlled trial. *PLoS One*.

[12] Chen R., Chen M., Kang M. (2010). The design and protocol of heat-sensitive moxibustion for knee osteoarthritis: a multicenter randomized controlled trial on the rules of selecting moxibustion location. *BMC Complementary and Alternative Medicine*.

[13] Chen R., Xie D. (2019). Construction and clinical application of the theoretical system of heat-sensitive moxibustion. *World Chinese Medicine*.

[14] Xiong J., Zhang W., Jiao L. (2015). Different warm sensations may induce different therapeutic effects in primary dysmenorrhea patients undergoing moxibustion treatment based on propensity score: a prospective cohort study. *Zhen Ci Yan Jiu*.

[15] Zhang B., Chen Y. Q., Zhou C. X., Chen R. X. (2020). Technical elements and clinical application of umbilical refining of heat-sensitive moxibustion. *Chinese Acupuncture & Moxibustion*.

[16] Wang Z. L. (1989). Principles of dysmenorrhea: based on clinical research of traditional Chinese medicine. *Journal of Electron Microscopy Technique*.

[17] Guan Q., Zhang H., Zhang S. (2021). Clinical study on heat-sensitive moxibustion combined with Chinese medicine point Application for primary dysmenorrhea of cold congealing and blood stasis type. *New Chinese Medicine*.

[18] Lin W. (2021). Clinical observation of thermosensitive moxibustion on primary dysmenorrhea of cold coagulation and blood stasis type. *Fujian University of Traditional Chinese Medicine*.

[19] Zhang Q., Cao Y., Chen B. (2020). Comparison of the effects of thermal moxibustion and traditional suspension moxibustion in the treatment of primary dysmenorrhea (cold and damp stagnation type). *Inner Mongolia Traditional Chinese Medicine*.

[20] Zhu L. (2020). Clinical research on the treatment of primary dysmenorrhea with Wenjing Zhitong decoction combined with heat-sensitive moxibustion. *Shandong University of Traditional Chinese Medicine*.

[21] Wang Y. (2020). Clinical efficacy of shao abdominal conveying blood stasis tang plus reduction combined with heat-sensitive moxibustion in the treatment of primary dysmenorrhea caused by cold clotting and blood stasis. *Fujian University of Traditional Chinese Medicine*.

[22] Li X. (2020). Evaluation of the effect of heat-sensitive moxibustion of Sanyinjiao plus ear acupressing beans for primary dysmenorrhea. *Journal of Shandong Medical High School*.

[23] Han Y. (2019). Clinical study of thermal moxibustion combined with acupuncture in the treatment of cold coagulation and blood tasis dysmenorrhea. *Changchun University of Traditional Chinese Medicine*.

[24] Wang M., Diao J. (2019). Clinical efficacy of combining Angelica Sinensis Four Inversions Tang with thermal moxibustion in the treatment of primary dysmenorrhea. *Jiangxi Traditional Chinese Medicine*.

[25] Li J., Gao Y., Zhao X. (2019). Clinical observation of heat-sensitive moxibustion in the treatment of primary dysmenorrhea. *Bright Chinese Medicine*.

[26] Ou Z. (2017). Clinical observation of heat-sensitive moxibustion on primary dysmenorrhea of cold and damp stagnation type. *Bright Chinese Medicine*.

[27] Ma H., Hou X., Wan G. (2016). The efficacy of heat-sensitive moxibustion in treating primary dysmenorrhea in the form of cold clotting and blood stasis. *Journal of Practical Chinese Medicine*.

[28] Xie H., Liu F., Jiao L., Chen R. (2016). Clinical efficacy of heat-sensitive moxibustion in the treatment of primary dysmenorrhea and common concomitant symptoms during menstruation. *Shi Zhen National Medicine*.

[29] Lu H., Lu J., Jin H. (2015). The efficacy of heat-sensitive moxibustion in treating primary dysmenorrhea in 40 cases. *Zhejiang Journal of Traditional Chinese Medicine*.

[30] Zhang W., Li H., Hu J. (2014). Correlation between moxibustion sensation and moxibustion efficacy of heat-sensitive moxibustion “Guan Yuan” point for primary dysmenorrhea. *Shi Zhen National Medicine*.

[31] Zhong X. (2014). A Controlled study on the efficacy of heat-sensitive moxibustion and Inter-ginger moxibustion in the treatment of primary dysmenorrhea with cold and damp stagnation. *Chengdu University of Traditional Chinese Medicine*.

[32] Li J., Wei D., Wang Y., Ding H., Wang Y. (2013). Clinical observation of thermal moxibustion for primary dysmenorrhea. *Jilin Traditional Chinese Medicine*.

[33] Nie R., Huang C., Li F., Fu Y., Li H. (2010). Clinical observation of heat-sensitive moxibustion for primary dysmenorrhea. *China Journal of Traditional Chinese Medicine Information*.

[34] Rao Y. (2009). 30 cases of primary dysmenorrhea treated with heat-sensitive point moxibustion therapy. *Jiangxi Traditional Chinese Medicine*.

[35] Zhang H., Fu Y., Zhang B. (2008). Clinical study of heat-sensitive sensitization moxibustion for primary dysmenorrhea. *Henan Traditional Chinese Medicine*.

[36] (2018). ACOG Committee Opinion No. 760: Dysmenorrhea and endometriosis in the adolescent. *Obstetrics & Gynecology*.

[37] Wehling M. (2014). Non-steroidal anti-inflammatory drug use in chronic pain conditions with special emphasis on the elderly and patients with relevant comorbidities: management and mitigation of risks and adverse effects. *European Journal of Clinical Pharmacology*.

[38] Harel Z. (2004). Cyclooxygenase-2 specific inhibitors in the treatment of dysmenorrhea. *Journal of Pediatric and Adolescent Gynecology*.

[39] Xie D., Liu Z., Hou X. (2013). Heat sensitisation in suspended moxibustion: features and clinical relevance. *Acupuncture in Medicine*.

[40] Chen R., Chen M., Kang M., Chi Z. H., Zhang B. (2010). Paying attention to the heat thermal sensitivity of moxibustion is the key for raising the curative effect. *Acupuncture Research*.

[41] Xiong J., Chen Y., Chen R. (2020). Professor CHEN Ri-xin’s academic thought and clinical application of “no allergy without any deficiency”. *Chinese Acupuncture & Moxibustion*.

[42] He W., Shi X. S., Zhang Z. Y. (2020). Discussion on the effect pathways of preventing and treating coronavirus disease 2019 by acupuncture and moxibustion from the regulation of immune inflammatory response. *Chinese Acupuncture & Moxibustion*.

[43] Zuo X., Shi Y., Li Y. (2016). Evaluation of the efficacy of gentle moxibustion in the treatment of functional dyspepsia with spleen qi deficiency. *World Latest Medicine Information*.

