# Supplementary material for: Curative Effect of Heat-sensitive Moxibustion on Primary Dysmenorrhea: A Meta-Analysis
Source: Evid Based Complement Alternat Med. 2022 Jul 30;2022:1281336. doi: 10.1155/2022/1281336 (PMC9356805; doi:10.1155/2022/1281336)
Supplement: Supplementary Materials — Subgroup analysis of the total effective rate was performed on Supplemental Figure 1. Subgroup analysis of the VAS score was performed on Supplemental Figure 2. Subgroup analysis of the symptom score was performed on Supplemental Figure 3. Subgroup analysis of the CMSS score was performed on Supplemental Figure 4. [file 1281336.f1.zip › 1281336.f1/Supplement figure4.pdf]

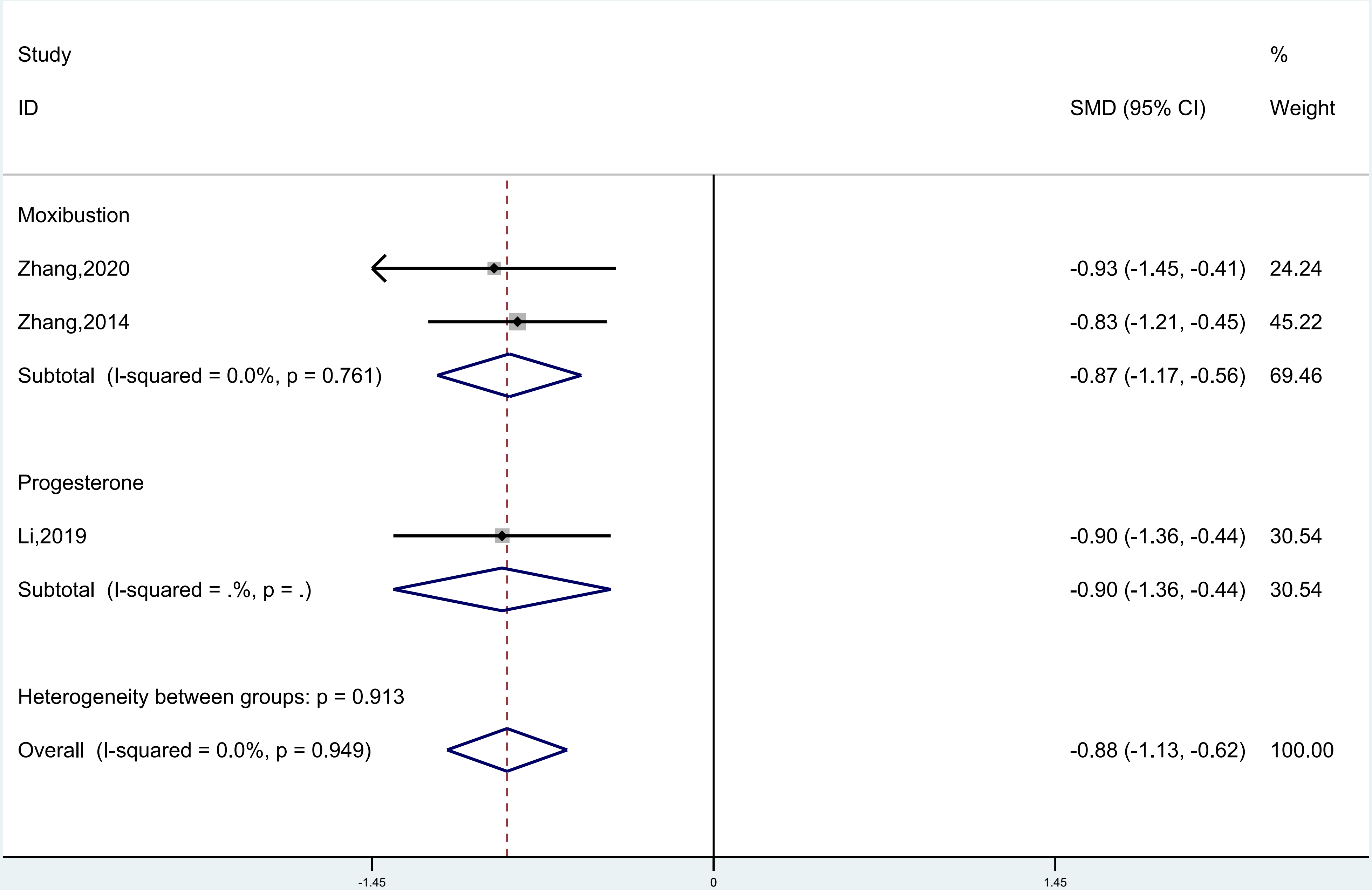
Study
ID
%
SMD (95% CI)
Weight
Moxibustion
Zhang,2020
-0.93 (-1.45, -0.41)
24.24
Zhang,2014
-0.83 (-1.21, -0.45)
45.22
Subtotal (I-squared = 0.0%, p = 0.761)
-0.87 (-1.17, -0.56)
69.46
Progesterone
Li,2019
-0.90 (-1.36, -0.44)
30.54
Subtotal (I-squared = .%, p = .)
-0.90 (-1.36, -0.44)
30.54
Heterogeneity between groups: p = 0.913
Overall (I-squared = 0.0%, p = 0.949)
-0.88 (-1.13, -0.62)
100.00
-1.45
0
1.45
